# Breast Cancer Prediction Using Rotation Forest Algorithm Along with Finding the Influential Causes

**DOI:** 10.3390/bioengineering12101020

**Published:** 2025-09-25

**Authors:** Prosenjit Das, Proshenjit Sarker, Jun-Jiat Tiang, Abdullah-Al Nahid

**Affiliations:** 1 Electronics and Communication Engineering Discipline, Khulna University, Khulna 9208, Bangladesh; prosenjitdas0929@gmail.com (P.D.); prosensarker4@gmail.com (P.S.); 2 Centre for Wireless Technology, CoE for Intelligent Network, Faculty of Artificial Intelligence & Engineering, Multimedia University, Persiaran Multimedia, Cyberjaya 63100, Selangor, Malaysia

**Keywords:** breast cancer, Optuna, rotation forest, wrapper method, ensemble voting classifier, counterfactual analysis

## Abstract

Breast cancer is a widespread disease involving abnormal (uncontrolled) growth of breast tissue cells along with the formation of a tumor and metastasis. Breast cancer cases occur mostly among women. Early detection and regular screening have significantly improved survival rates. This research classifies breast cancer and non-breast cancer cases using machine learning algorithms based on the Breast Cancer Coimbra dataset by optimizing the classifier performance and feature selection methodology. In addition, this research identifies the influential features responsible for BC classification by using diverse counterfactual explanations. The Rotation Forest classifier algorithm is used to classify breast cancer and non-breast cancer cases. The hyperparameters of this algorithm are optimized using the Optuna optimizer. Three wrapper-based feature selection techniques (Sequential Forward Selection, Sequential Backward Selection, and Exhaustive Feature Selection) are used to select the most relevant features. An ensemble environment is also created using the best feature subsets of these methods, incorporating both soft and hard voting strategies. Experimental results show that the hard voting strategy achieves an accuracy of 85.71%, F1-score of 83.87%, precision of 92.85%, and recall of 76.47%. In contrast, the soft voting strategy obtains an accuracy of 80.00%, F1-score of 77.42%, precision of 85.71%, and recall of 70.59%. These findings demonstrate that hard voting achieves noticeably better performance. The misclassification outcomes of both strategies are explored using Diverse Counterfactual Explanations, revealing that BMI and Glucose values are most influential in predicting correct classes, whereas the HOMA, Adiponectin, and Resistin values have little influence.

## 1. Introduction

**Figure 1:**
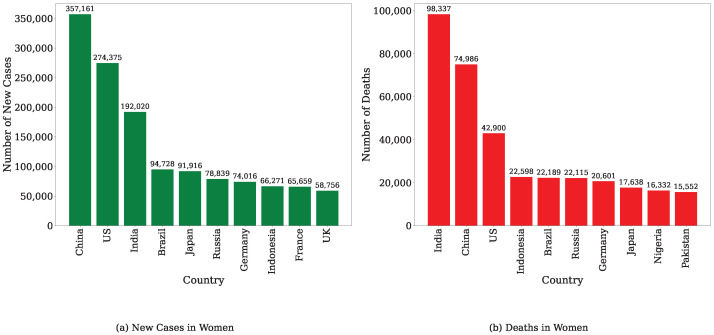
This figure shows the information of the ten countries with the highest breast cancer incidences and deaths in women.

Breast cancer (BC) is a type of cancer in which malignant cells begin to develop inside the milk ducts and/or the milk-producing lobules of the breast [1]. Among all types of cancers, breast cancer is one of the most prevalent. BC has been identified as the most common and fatal cancer among women in 157 out of 185 countries as of 2022 [2]. The occurrence of BC in women is 100 times higher than in men [3]. BC primarily affects women and can occur at any stage of puberty, although its incidence becomes more frequent with advancing age. In 2022, approximately 2.3 million women were diagnosed with breast cancer, with 670,000 deaths reported globally [2]. According to global cancer statistics published in 2022, based on the number of new cases, BC ranks second in incidence and fourth in mortality among all cancers [4]. Furthermore, Figure 1 illustrates the ten countries with the highest incidence and mortality rates of breast cancer in womenaccording to the World Cancer Research Fund’s 2022 report [5].

When cancer cells become invasive and spread to nearby lymph nodes or distant organs (metastasis), BC can become life-threatening. It is seen as fatal and very dangerous to have metastasis. Early detection of BC has saved thousands of women’s lives by not allowing the cancer to advance past the early stages of the disease. Regular mammograms, breast self-tests, and awareness of symptoms lead to early BC detection, which improves survival rates and treatment. Traditional technologies such as mammography, biopsies, magnetic resonance imaging (MRI), ultrasound, and clinical examinations take a long time, are costly, and can involve a human error factor. In the past decade, machine learning (ML) has become a powerful technique for directly improving breast cancer diagnosis, classification, and prognosis from complex biomedical data. ML models learn patterns from factual patient data with which to make predictions on future cases. These models use large datasets of imaging data, blood biomarkers (as in the Breast Cancer Coimbra dataset), genetic information, and clinical history to spot minute cancer pointers. Algorithms such as Support Vector Machine (SVM), Random Forest, Artificial Neural Network (ANN), and XGBoost have been used successfully by researchers to accurately distinguish benign and malignant cases.

In addition, ML is helpful for feature selection, determining the most relevant biological indicators of cancer progression that might help doctors to formulate personalized treatment plans. As data quality, processing capacity, and algorithms continue to improve, machine learning will increasingly transform the early detection and treatment of breast cancer. In addition to image data, there has been a growing interest in the use of blood-based biomarkers for the classification of BC, as it has the advantages of being non-invasive and more accessible in practice. Datasets with numbers of instances and attributes, such as the Breast Cancer Coimbra and Breast Cancer Wisconsin (BCW) datasets, have been used to assess the performance of ML algorithms on such biomarkers. Assegie used the BCW dataset for classifying breast cancer events using ML models such as the K-Nearest Neighbor (KNN) classifier [6]. While the BCW dataset has mostly been employed in studying the morphology of breast cells, recent investigations have taken advantage of the BCC dataset, focusing on the information about several metabolic and hormonal markers it provides [7]. This dataset has been shown to perform well using various algorithmic models.

For instance, Polat et al. used the AdaBoostM1 classifier with MAD normalization method, yielding a 75% classification accuracy, whereas a hybrid approach achieved 91.37% accuracy [8]. Li et al. applied the Random Forest classifier and Support Vector Machine (SVM), achieving 74.3% and 71.4% accuracy, respectively [9]. On the other hand, in [10] traditional classifiers such as SVM and KNN achieved accuracy of 71% and 81%, respectively. Abdulkareem et al. used an optimized KNN, achieving an accuracy of 87.50% [11]. Previous studies have not shown any feature reduction techniques, and no misclassification analysis has been conducted. As well, no mechanism for correcting wrong predictions has been introduced. To address these limitations, we have organized our contributions as follows:

- We use an optimization algorithm to select the best hyperparameters for the classifier model.
- This study implements wrapper-based feature selection techniques, using the optimized parameters to identify the most relevant features.
- We analyze misclassified and correctly classified data using Diverse Counterfactual Explanations (DiCE) to provide interpretability and insights into the model’s decisions.

The introductory information of this experiment has been presented in the Introduction section; in the rest of the paper, the methodology of the study is detailed in Section 2; Section 3 provides a comprehensive explanation of the results along with their analysis along with a general discussion based on the findings; finally, the research is concluded in Section 4.

## 2. Methodology

**Figure 2:**
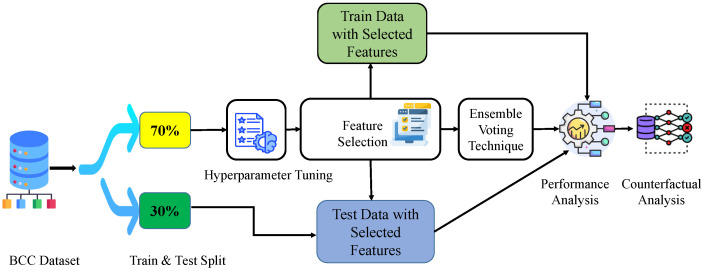
Representation of methodology architecture.

This research analysis evaluates a classification task to identify affected BC and non-BC patients using the BCC dataset. The BCC dataset is labeled based on affected BC and non-BC patients; therefore, this classification task is implemented using a supervised machine-learning approach. Additionally, the research has performed data exploration, hyperparameter tuning through optimization, feature selection, and feature prioritization. To perform the classification task, the Rotation Forest (RF) algorithm is applied. Optuna hyperparameter tuning is used to find the best parameters of the RF algorithm. The wrapper-based feature selection technique operates to find the best feature subsets. Three wrapper-based feature selection techniques are applied, including Sequential Forward Selector (SFS), Sequential Backward Selector (SBS), and Exhaustive Feature Selector (EFS). Moreover, a feature subset-based Ensemble Voting Classifier (Soft and Hard Voting) is used, in which different RF models are trained to improve evaluation performance. For evaluation, the parameters of accuracy, F1 score, precision, and recall are considered. Lastly, Diverse Counterfactual Explanations (DiCE) are utilized. The overall classification methodology has been represented in Figure 2.

### 2.1. Dataset

The BCC dataset has been gathered from the University Hospital of Coimbra, Portugal, and is available via the UCI Machine Learning Repository. The dataset was retrieved on 25 April 2025, and can be accessed at https://doi.org/10.24432/C52P59. It contains information on 116 female patients with nine clinical and biochemical features: age, BMI, glucose, insulin, HOMA, leptin, adiponectin, resistin, and MCP-1, along with a target feature serving as the classification label [7]. The classification label consists of the values 1 and 2, where 1 represents Non-BC and 2 represents BC. Out of the 116 patients, 64 have been diagnosed with BC and 52 with Non-BC. All features in the BCC dataset are numerical, and Table 1 provides a summary of the feature descriptions [7].

**Figure 3:**
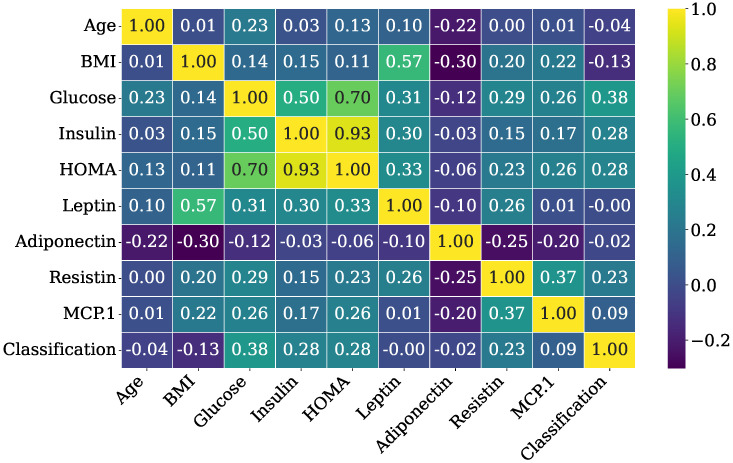
Visual representation of correlation among different features of the BCC dataset.

Additionally, Figure 3 presents the correlation matrix of the BCC dataset, displaying all pairwise Pearson correlation coefficients among the variables. Very strong positive correlations can be observed between Insulin and HOMA (0.93) as well as between Glucose and HOMA (0.70). Glucose also shows the highest correlation (0.38) with the classification target, indicating its potential importance in predicting breast cancer.

### 2.2. Optuna Hyperparameter Optimization

Optuna is a powerful and flexible hyperparameter optimization framework aimed at automating the tuning process for ML and DL models. It is open source and designed for ease of use. It was created by Takuya Akiba and colleagues with an emphasis on flexibility, allowing users to set their objective functions, as well as efficiency, allowing for sophisticated sampling and pruning methods to search through complicated hyperparameter spaces [12]. In contrast to traditional grid and random search for hyperparameter tuning, Optuna uses intelligent sampling of the hyperparameter search space via sophisticated algorithms such as the Tree-structured Parzen Estimator (TPE) [13]. Its popularity is due to its simplicity, the fact that it discards failed trials, and because it is implemented in many very popular ML libraries such as Random Forest, XGBoost, LightGBM, and Rotation Forest.

In this study, we used the F1-score as the objective function. In addition, the model has been trained on a fixed training set, and its predictions have been evaluated on the same training data using the F1-score. Each time, Optuna selects key hyperparameters of the RF classifier and provides the relatively best parameters to maximize the F1-score. In this research work, Optuna was utilized with the TPE as a sampler, as proposed by Bergstra et al. TPE is used to perfectly explore the hyperparameter space with a fixed seed for consistency [2]. In this case, a total of 50 trials were conducted. Equation (1) [13] shows the basic structure of the TPE, ensuring the proper optimization of Expected Improvement.

(1) $${\mathrm{EI}}_{y^{*}}\left(x\right)=\frac{\gamma \;\ell (x)}{\gamma \;\ell (x)+(1-\gamma )\;g(x)}$$

Here, $\ell \left(x\right)=p\left(x|y<y^{*}\right)$ and $g\left(x\right)=p\left(x|y\geq y^{*}\right)$ are densities estimated using Parzen estimators over the best and remaining observations, respectively, while $\gamma =p(y<y^{*})$ is the quantile used to split the observations.

Although Optuna supports early-stopping methods via pruning strategies, in our study we deliberately did not incorporate pruning because we wanted to ensure the same number of evaluations in all experiments to avoid bias. The optimization objective was evaluated on the training set.

In this context, the number of estimators, minimum group, maximum group, and remove proportion hyperparameters of RF were tuned, as explained in Table 2. In addition, we used a fixed random state parameter to control the randomness.

### 2.3. Classification Model

**Figure 4:**
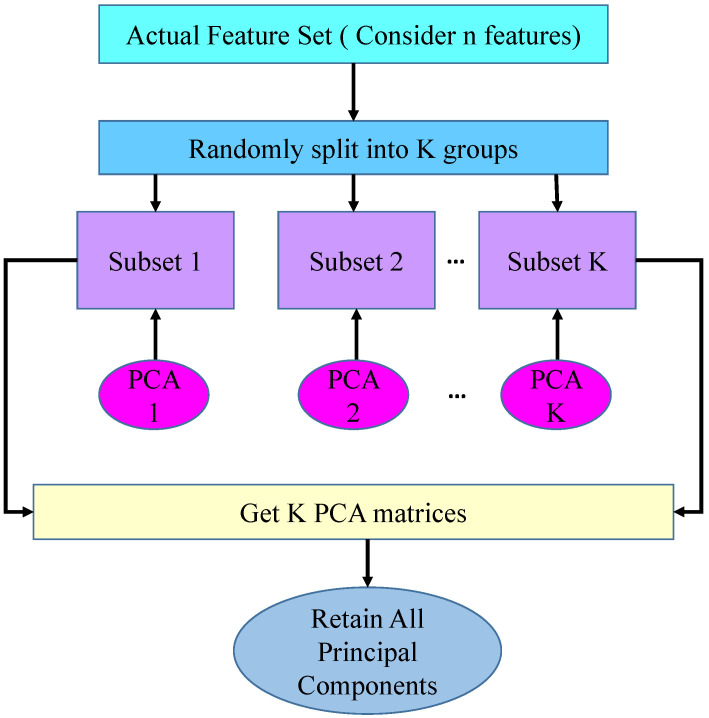
Representation of the working process of PCA in the RF classifier.

RF is an ensemble learning classification method that computes many so-called decision trees derived from rotations of the feature space [14]. To ensure diversity, the base classifiers were trained on subsets of the features obtained by applying Principal Component Analysis (PCA). The goal in this approach is to maximize diversity in the ensemble while ensuring accuracy for each classifier. RF also randomizes features by rotating feature space, allowing for overfitting reduction and better generalization. In RF, randomness is generated by randomly dividing the feature space into subsets and utilizing bootstrapping. Randomness is configured in RF using the random state parameter, in which the random state parameter takes any random number to control the randomness. All the principal components are retained to preserve all information and avoid information loss [14]. In RF, all the principal components are retained, not used for dimensionality reduction. In this context, the main goal of PCA is to enable full-axis rotation and maintain classifier diversity across the ensemble. For a base classifier, the PCA working method diagram is represented in Figure 4. This process is repeated for all base classifiers. The final prediction of the RF classifier is made by majority vote among the base classifiers. Essentially, the base classifier individually predicts class labels, with the final prediction taken based on the highest number of votes from all base classifiers. RF performs competitively with other ensemble methods such as Bagging and AdaBoost [14].

### 2.4. Justification for Choosing Algorithm

We have chosen the RF classifier algorithm because of its enhanced diversity through feature rotation. RF uses PCA, in which the purpose of PCA is to preserve classifier diversity throughout the ensemble and allow full-axis rotation. For this reason, we have selected the RF algorithm.

### 2.5. Hyperparameter Range and Justification

In this study, we have optimized four hyperparameters for the RF classifier: the number of estimators, the minimum group size, the maximum group size, and the remove proportion. Table 2 presents the boundary conditions for these hyperparameters. The number of estimators has been varied in the range of 50 to 300. The minimum group size has been constrained between 2 and 5. Because the maximum group size depends on the selected minimum group size, its range has been set from the chosen minimum group value up to 5. Lastly, the remove proportion has been explored in the range of 0.1 to 0.7.

The default hyperparameter settings for the Rotational Forest classifier are n_estimators=200, min_group=3, max_group=3, and remove_proportion=0.5 [15]. To ensure comprehensive exploration and avoid missing potentially better configurations, we have defined broader search ranges around these defaults. Specifically, the number of estimators was varied from 50 to 300, allowing for both smaller and larger ensemble sizes than the default. The minimum and maximum group sizes were expanded to span from 2 to 5, capturing more flexible subsample grouping. Lastly, the removal proportion was adjusted between 0.1 and 0.7. This extended search space facilitates the optimizer’s ability to identify more optimal hyperparameter values beyond the default settings.

### 2.6. Feature Selection Techniques

In this study, the following three wrapper-based feature selection techniques have been used:

**(i) SFS:** SFS is a wrapper-based feature selection method in machine learning. It starts with an empty feature set, gradually building the setup by adding one feature at a time. Model performance is assessed at each step of the way, repeating until some stopping criterion is achieved [16]. For SFS, the detailed explanation is represented in Algorithm 1.
**Algorithm 1** Sequential Forward Feature Selection Algorithm [17]**Require:** Set of features $X=\{x_{0},x_{1},\ldots ,x_{n}\}$, total number of features *n*, target subset *d***Ensure:** Suboptimal feature subset $Y_{\mathrm{subopt}}$ of size *d*  1: $Y_{\mathrm{subopt}}\leftarrow \emptyset$  2: **for **j=1 to *d* **do**  3:    $x\leftarrow \arg\max_{x_{i}\in X\setminus Y_{\mathrm{subopt}}}J\left(Y_{\mathrm{subopt}}\cup \left\{x_{i}\right\}\right)$  4:    $Y_{\mathrm{subopt}}\leftarrow Y_{\mathrm{subopt}}\cup \left\{x\right\}$  5: **end for**

**(ii) SBS:** SBS is another wrapper-based feature selection algorithm that is quite similar to SFS. The difference is in the procedure; while SBS removes features, SFS uses an additive approach [18]. Algorithm 2 presents how SBS works.
**Algorithm 2** Sequential Backward Feature Selection Algorithm [17]**Require:** Set of features $X=\{x_{0},x_{1},\ldots ,x_{n}\}$, total number of features *n*, target subset *d***Ensure:** Suboptimal feature subset $Y_{\mathrm{subopt}}$ of size *d*  1: $Y_{\mathrm{subopt}}\leftarrow X$  2: **for **j=1 to n−d **do**  3:    $x\leftarrow \arg\min_{x_{i}\in Y_{\mathrm{subopt}}}J\left(Y_{\mathrm{subopt}}\setminus \left\{x_{i}\right\}\right)$  4:    $Y_{\mathrm{subopt}}\leftarrow Y_{\mathrm{subopt}}\setminus \left\{x\right\}$  5: **end for**

**(iii) EFS:** EFS is a wrapper-based feature selection method and a brute-force method that evaluates all combinations of features in search of the subset that produces the best model performance. It explores all combinations of k features, where k varies from a minimum number to a maximum number of features. This approach has the guarantee of selecting optimal optimal-performing subset based on the evaluation metric used (e.g., accuracy, F1-score). Unfortunately, it is costly and is not feasible when there are many features. Thus, it is more appropriate for small to moderate-sized feature spaces [19]. Algorithm 3 represents the procedure for EFS.

After feature selection, we have employed separate pipelines with different subsets of features. Each pipeline combines selected features with an RF classifier model. These pipelines have been integrated into an ensemble voting classifier to enhance the model’s performance. In addition, we have applied hard and soft voting strategies individually and analyzed the results. Hard voting encompasses class labels based on the majority-rule scheme, while soft voting averages the estimated probabilities together, yielding a smooth decision surface. Ensemble voting has been adopted on the assumption that a selection of different classifiers can be better understood in generalization and stability.
**Algorithm 3** Exhaustive Feature Selection Algorithm [17]**Require:** Set of features *X*, total number of features *n*, target subset size *d*, set of all feature subsets *F* of size *d***Ensure:** Optimal feature subset $Y_{\mathrm{opt}}$ of size *d*  1: $Y_{\mathrm{opt}}\leftarrow \emptyset$  2: $G_{\mathrm{opt}}\leftarrow -\infty$  3: **for all** Yi∈F={Y0,Y1,…,Yk}∣k=nd **do**  4:    $G_{i}\leftarrow J\left(Y_{i}\right)$                                        ▹ Evaluate the subset using criterion function *J*  5:    **if** $G_{i}>G_{\mathrm{opt}}$ **then**  6:        $Y_{\mathrm{opt}}\leftarrow Y_{i}$  7:        $G_{\mathrm{opt}}\leftarrow G_{i}$  8:    **end if**  9: **end for**

### 2.7. Evaluation Criteria

This section presents conventional evaluation criteria for assessing the model’s performance using the standard classification metrics of accuracy, F1-score, precision, and recall, all derived from the confusion matrix. The Confusion Matrix (CM) serves as a fundamental tool in this context. It contains four categories based on the predicted and actual classes. These four categories are as follows:

- True Positive (TP): Correctly predicted the positive class.
- False Positive (FP): Incorrectly predicted the positive class.
- True Negative (TN): Correctly predicted the negative class.
- False Negative (FN): Incorrectly predicted the negative class.

The classification metrics are mathematically calculated as shown below.

(2) $$\begin{array}{cc} \mathrm{Accuracy} & =\frac{TP+TN}{TP+TN+FP+FN} \end{array}$$

(3) $$\begin{array}{cc} \mathrm{Precision} & =\frac{TP}{TP+FP} \end{array}$$

(4) $$\begin{array}{cc} \mathrm{Recall} & =\frac{TP}{TP+FN} \end{array}$$

(5) $$\begin{array}{cc} \mathrm{F1-Score} & =\frac{2\cdot \mathrm{Precision}\cdot \mathrm{Recall}}{\mathrm{Precision}+\mathrm{Recall}} \end{array}$$

These classification metrics were used for each evaluation task in this research. The evaluation task was computed on the training and testing sets. Additionally, the Receiver Operating Characteristic (ROC) and Area Under the Curve (AUC) are analyzed in this study.

ROC is a graphical representation used to visualize the performance of binary classification at various threshold settings. The ROC curve is plotted as the True Positive Rate (also known as the sensitivity or recall) against the False Positive Rate (also known as the fallout or 1-specificity).

AUC is a performance metric that quantifies the classifier’s ability to discriminate between classes. A value of AUC closer to 1 represents better performance. The mathematical presentation of ROC and AUC is shown below.

(6) $$\begin{array}{cc} \mathrm{False}\;\mathrm{Positive}\;\mathrm{Rate}\;\left(\mathrm{FPR}\right) & =\frac{FP}{FP+TN} \end{array}$$

(7) $$\begin{array}{cc} \mathrm{AUC} & =\int_{0}^{1}\mathrm{TPR}\left(\mathrm{FPR}\right)\;d\left(\mathrm{FPR}\right) \end{array}$$

### 2.8. Diverse Counterfactual Explanations

Diverse Counterfactual Explanations (DiCE) is a method that produces multiple, diverse, and realistic counterfactuals to explain predictions of ML models [20]. Rather than a single explanation, DiCE exposes users to a variety of alternative possible scenarios that would have led to a different outcome in the model, making it appropriate for action-oriented decisions. It combines balance in prediction accuracy, small changes in features, and diversity to offer useful and realistic explanations. DiCE is model-agnostic and has proven useful in interpretable domains such as healthcare and finance. Mothilal et al. proposed this method in 2020 to improve the transparency and fairness of ML systems [20]. The optimization problem solved by DiCE is formulated in Equation (8).

(8) $$C\left(z\right)=\arg\min_{c_{1},\ldots ,c_{k}}\frac{1}{k}\sum_{j=1}^{k}L_{\mathrm{cls}}\left(h\left(c_{j}\right),t\right)+\frac{\alpha }{k}\sum_{j=1}^{k}D\left(c_{j},z\right)-\beta \cdot V\left(c_{1},\ldots ,c_{k}\right)$$

Here, $L_{\mathrm{cls}}$ ensures that each counterfactual $c_{j}$ aligns with the target class *t*, D maintains proximity to the original input z, and V encourages diversity. The parameters α and β balance closeness and diversity. Thus, DiCE generates diverse and realistic counterfactuals to explore alternate model outcomes [20].

In this study, DiCE has been chosen over SHAP and LIME because they do not directly predict how much the inputs must change to produce another outcome. In this context, DiCE can predict how much the input value must change to generate a new outcome.

## 3. Results

In this research, the Results section has been divided into three parts: Wrapper-Based Feature Selection Method and Hyperparameter Output, Ensemble Voting Classifier (Soft and Hard Voting) Analysis, and Diverse Counterfactual Explanations on Misclassified Data. The first part elaborates the best feature set based on the accuracy of each wrapper case using corresponding hyperparameter values for the RF classifier. The second part explains the use of the ensemble voting classifier to obtain better outcomes and analysis. Lastly, the third part analyzes the application of DiCE to the misclassified data obtained from the ensemble classifier. This final subsection mainly explores how much of a patient’s information needs to change in order to result in a different outcome or deviate from the actual outcome.

### 3.1. Wrapper-Based Feature Selection Method and Hyperparameter Output

**Figure 5:**
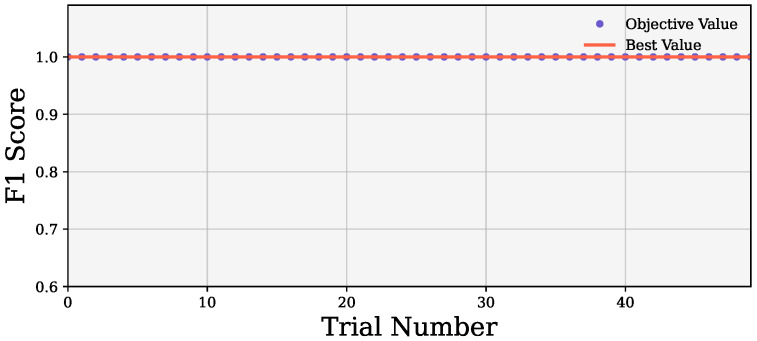
Representation of cost function.

All three wrapper methods were optimized with Optuna before evaluation. We used 50 trials in Optuna to extract the best hyperparameter set. During the optimization, the models gained a perfect cost function of 1 from the first trial, as shown in Figure 5. Table 3 shows the selected hyperparameter set. Optuna returned the best value of the number of estimators of 144, min and max group of 5, and remove proportion of 0.5391963650868431.

#### 3.1.1. Cross-Validation Performance

In addition, we observed the 5-fold cross-validation mean result before feature selection. In this context, we have used the hyperparameters for the RF classifier selected by the Optuna optimizer. In the 5-fold cross-validation context, the RF classifier achieved a mean accuracy of 73.99%, a mean F1-score of 67.34%, mean precision of 70.48%, and recall of 68.11%. The results are presented in Table 4.

#### 3.1.2. Wrapper-Based Feature Selection Performance

The parameters achieved from the Optuna result were used for each wrapper-method feature selection case and effectively determined the best features. Using the selected features, we evaluated the classification metrics of accuracy, F1-score, precision, and recall for SFS, SBS, and EFS.

**(i) SFS:** The SFS wrapper method selected a feature subset containing Age, BMI, Glucose, Adiponectin, and Resistin, which enabled the RF classifier to achieve an accuracy of 85.71% and F1-score of 83.87%. The model also secured a high precision of 92.85% and a recall of 76.47%, indicating the usefulness of SFS in carrying more informative features. These results reveal that SFS contributes to improved classification performance through optimal feature selection.

**(ii) SBS:** SBS chose a feature subset including Age, Glucose, HOMA, Adiponectin, and Resistin; however, the resulting model performance declined. The RF classifier achieved only 68.57% accuracy and 68.57% F1-score, with a precision of 66.66% and recall of 70.58%. This outcome suggests that SBS may retain less pertinent features.

**(iii) EFS:** EFS selected a similar optimal feature subset as SFS, resulting in corresponding classification outcomes. The RF classifier achieved 85.71% accuracy, 83.87% F1-score, 92.85% precision, and 76.47% recall using the selected features. Among the three methods (SFS, SBS, and EFS), Age, Glucose, Adiponectin, and Resistin emerged as common features, indicating their significant impact on the models. The results of each wrapper-method are presented in Table 4.

### 3.2. Ensemble Voting Classifier (Soft and Hard Voting) Analysis

**Figure 6:**
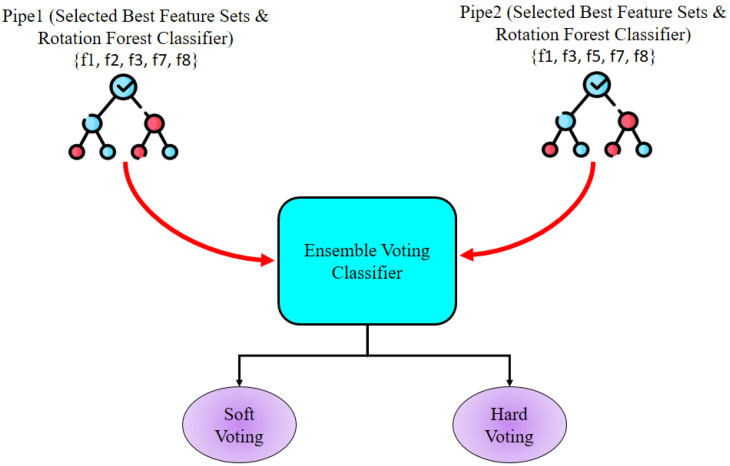
Ensemble Voting Classifier working process diagram used for this research.

In this research, ensemble learning techniques are used to intensify the classification performance by leveraging the strengths of multiple RF classifiers. Two distinct feature subsets were selected from the three wrapper-based feature selection techniques. Because SFS and EFS provided the same results and same feature subset, one feature subset was chosen from both and another was taken from SBS. For this ensemble learning approach, two pipelines were created, denoted Pipe1 and Pipe2, with each pipeline containing a distinct feature subset and RF classifier. The RF classifiers were parameterized using the optimal hyperparameters, and the Pipe1 and Pipe2 Ensemble Voting Classifier approaches were employed. Finally, soft and hard voting strategies were applied. The overall Ensemble Voting Classifier system is represented in Figure 6. For soft and hard voting, both cases assigned higher influence to Pipe1 before training.

Table 5 presents the comparative performance of the ensemble methods for hard voting and soft voting in terms of the most important evaluation metrics for both the training and test cases. In the training case, soft voting and hard voting both yielded 100% accuracy, F1-score, precision, and recall, which indicates that the classifier model performed better on seen data. In the test case, the hard voting method performed better with an accuracy of 85.71%, F1-score of 83.87%, and recall of 76.47%. It also showed better precision of 92.86%, indicating a lower rate of false positives. Soft voting continued to be competitive, with 80.00% accuracy, 77.42% F1-score, 85.71% precision, and 70.59% recall. These outcomes generally point towards hard voting offering more stable classification ability.

The soft and hard voting strategies were also used to explore their effectiveness and performance in terms of how much they varied. We obtained two identical feature subsets among the three wrapper-based feature selection techniques. In this case, we chose unique feature subsets as well as an identical one which was used to make the pipeline. We created two pipelines, as the two feature subsets are identical; if had we created three pipelines, then these two would be more influential, and the results might vary according to the identical features. To avoid this, we selected only one out of the two identical feature subsets.

**Figure 7:**
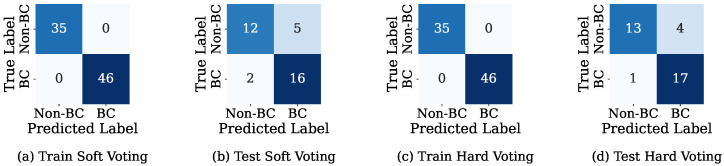
Confusion matrix for soft and hard voting.

Figure 7 presents the confusion matrices for both soft and hard voting ensemble classifiers in distinguishing between the Non-BC (Non-Breast Cancer) and BC (Breast Cancer) classes for both the training and test cases. In the training case, both soft voting and hard voting correctly classified BC and Non-BC, indicating that the ensemble voting classifier models were able to effectively fit the training data without any misclassifications. The hard voting method correctly classified more cases (17 BC and 13 Non-BC) than the soft voting model (16 BC and 12 Non-BC). Additionally, by attaining reduced misclassification rates, the hard voting classifier showed enhanced overall prediction reliability. These results are supported by the superior performance metrics of the hard voting method.

**Figure 8:**
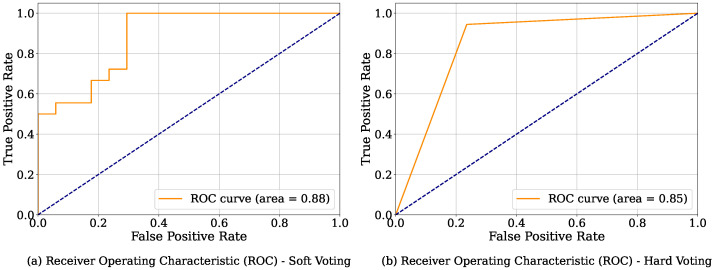
ROC curves comparison of soft and hard voting.

Figure 8 illustrates the Receiver Operating Characteristic (ROC) curves for the soft and hard voting ensemble classifiers. The Area Under the Curve (AUC) for soft voting is slightly higher, designating a better overall ranking performance. Although hard voting yields higher accuracy, soft voting demonstrates stronger discriminative ability across all threshold levels. The ROC curves for both soft and hard voting cases show that soft voting achieves a higher AUC of 88% compared to hard voting with an AUC of 85%, which highlights the former’s better discriminatory power. The ROC curves were developed using the test cases. The performance gap between soft and hard voting may have occurred due to their different mechanisms, as hard voting predicts the final class by majority vote whereas soft voting predicts the final class using the average predicted probabilities. This suggests that soft voting was able to generalize better under varied classification thresholds.

The misclassified examples for the soft and hard voting ensemble classifiers are shown in Table 6. Key characteristics such as Age, BMI, Glucose, HOMA, Adiponectin, Resistin, and the label transitions shown in the “Label Pair” column have been included in each table. Hard voting classifies five cases incorrectly, while soft voting classifies seven cases incorrectly. Both cases struggle to correctly identify class 1 (Non-BC), which they tend to predict as class 2 (BC). Notably, the instances indexed 18, 24, 30, 31, and 114 were misclassified by both classifiers. The reduced number of errors demonstrates the relatively higher robustness of hard voting in classification accuracy.

Table 7 presents a comparative performance analysis between the proposed models and other classifiers. We compared our work with the XGBoost, LightGBM, and CatBoost classifier models. The Extreme Gradient Boosting (XGBoost) classifier model yielded promising results, obtaining an accuracy of 82.86%, and the model maintained scores across key evaluation metrics, with F1-score, precision, and recall all at 83.33%, highlighting its reliable and balanced predictions. The Light Gradient-Boosting Machine (LightGBM) classifier achieved an accuracy of 80%, F1-score of 78.79%, precision of 81.25%, and recall 76.47%. In addition, the CatBoost classifier achieved an accuracy of 77.14%, F1-score of 75%, precision of 80%, and recall of 70.59%.

In this context, Our Work-1, using the RF classifier 5-fold cross-validation technique, achieved a moderate accuracy of 75.43% and moderate precision of 75%, whereas its F1-score and recall results were 71.61% and 70%, respectively. The Our Work-2 model applies SFS/EFS (RF) and SBS (RF); the former demonstrated excellent performance, with accuracy of 85.71%, precision of 83.87%, recall of 92.86%, and an F1-score of 76.47%, while the latter produced slightly worse results, with an accuracy of 68.57%, precision of 68.57%, recall of 66.66%, and F1-score of 70.58%. The Our Work-3 model utilizes two ensemble voting classifiers; hard voting yielded results similar to those of SFS/EFS, with an accuracy of 85.71%, precision of 83.87%, recall of 92.86%, and F1-score of 76.47%, while soft voting showed a medium level of performance, with accuracy of 80%, precision of 77.42%, recall of 85.71%, and an F1-score of 70.59%.

### 3.3. DiCE Analysis

Next, we analyzed the misclassified and correctly classified instances using DiCE.

#### 3.3.1. DiCE on Misclassified Instances

Table 6 provides the misclassification information for the soft and hard voting ensemble classifiers. Soft voting incorrectly predicted seven instances, while hard voting incorrectly predicted five instances. Five of the instances were misclassified by both classifiers. The instances indexed 18, 24, 30, 31, and 114 were incorrectly classified with both soft and hard voting, while the two instances indexed 26 and 100 were incorrectly classified by the soft voting classifier. DiCE was applied to describe the misclassified data.

For Index 26, the instance was misclassified as class 2 (BC), while its actual class label is 1 (Non-BC). As shown in Table 8, the generated counterfactuals specify that decreasing the Glucose level and increasing Resistin or BMI successfully changed the predicted outcome to the correct class. Thus, these features played a crucial role in influencing the model’s decision boundary. The first counterfactual of index 26 provided a significant reduction of Glucose levels from 106 to 89 and a substantial increase of Resistin levels from 11.78 to 58.17, which flipped the prediction to the actual class 1 (Non-BC). Additionally, the actual class 1 (Non-BC) was predicted by the second counterfactual of index 26, which shows a decrease in BMI levels from 38.58 to 32.32 and a decrease in glucose levels from 106 to 75. The last counterfactual analysis of index 26 explains the notable increase of BMI levels from 38.58 to 68 and the increase of Adiponectin levels from 4.67 to 7.54, and predicts the actual class 1 (Non-BC).

For Index 100, the instance was mistakenly predicted as class 1 (Non-BC) despite belonging to class 2 (BC). According to Table 8, increasing HOMA, Adiponectin, or Resistin or substantially raising Glucose results in the model switching its prediction to class 2. This highlights the importance of these metabolic parameters in the classification outcome. The first and second counterfactuals of index 100 provide information on a meaningful increase of HOMA levels from 0.65 to 14.98 and 15.50. In addition, the first counterfactual of index 100 changes the Resistin levels from 18.36 to 49.78, while the second counterfactual of index 100 significantly changes the Adiponectin levels from 7.65 to 23.90, overturning the correct prediction of class 2 (BC). Interestingly, the third counterfactual of index 100 considerably changes the BMI levels from 28.65 to 29.89 and sufficiently changes the Glucose levels from 88 to 189, resulting in the instance being correctly predicted as class 2 (BC).

For Index 24, the instance is misclassified as class 2 (BC), although it truly belongs to class 1 (Non-BC). The counterfactuals in Table 9 reveals that adjusting BMI or Adiponectin or reducing Glucose corrects the misclassification. These features exhibit a strong influence on the model’s output. The first and second counterfactuals of index 24 moderately increase the BMI level from 30.48 to 35.81 and 36.49, while the third counterfactual shows a slight decrease in BMI level from 30.48 to 28.86. In addition, the first counterfactual of index 24 sufficiently increases the Adiponectin level from 9.73 to 33.06, whereas the third counterfactual changes the Glucose level from 90 to 69. The general counterfactual analysis for index 26 achieves the correct prediction of class 1 (Non-BC).

For Index 31, the model wrongly predicts class 2 (BC) instead of the actual class 1 (Non-BC). The generated counterfactual shows that lowering Glucose and modifying Resistin or HOMA leads to a class change. These findings highlight the model’s sensitivity to glucose regulation markers. In this case, all counterfactuals of Index 26 show relatively reduced Glucose levels. In addition, the second counterfactual of Index 31 shows slightly changed Resistin levels, from 10.26 to 8.78; meanwhile, the third counterfactual of Index 31 shows expanded HOMA levels from 2.53 to 14.09. Finally, the overall analysis helps to predict the actual class 1 (Non-BC).

The DiCE analysis of cases incorrectly classified reveals that BMI and Glucose were the most important characteristics for accurate BC prediction, whereas HOMA, Adiponectin, and Resistin had less of an impact.

#### 3.3.2. DiCE on Correctly Classified Instances

In this context, we have observed how the correctly predicted class switches into another class. We analyzed only two instances in these cases.

Table 10 provides the actual case and counterfactual case of two samples (Index 1 and 2) initially classified as Class 1 (Non-BC). Notably, particular changes in features can lead to a change in prediction to Class 2; counterfactual examples have been provided. It is interesting to note that the features such as Glucose, HOMA, Resistin, and Adiponectin play an important role in flipping the class.

From the DiCE analysis, we have observed that the BMI, Glucose, HOMA, Resistin, and Adiponectin features play an important role in switching the class. In this context, they are important features of this perspective. In previous studies, one of the best indicators of BC is fasting glucose, and elevated glucose levels encourage it [21,22]. BC cells (MDAMB231, SKBR3, and MCF-7 cells) proliferate considerably more readily when glucose levels are higher than 25 mmol/L [23]. Another significant biomarker that promotes EMT and stemness by increasing BC metastasis is the protein Resistin, which is largely mediated by CAP1 [24]. Resistin is regarded as a critical risk factor and is considerably greater in BC patients (5.23 ± 6.9 ng/mL) than in Non-BC controls (1.46 ± 2.0 ng/mL) [25,26]. According to earlier research, there is an inverse relationship between BC and the hormone Adiponectin, which is released by adipocytes [27]. Numerous studies have demonstrated a correlation between a higher risk of BC and being overweight or obese, which indicates a high BMI [28].

## 4. Conclusions

This research has classified BC and non-BC patients by applying ML algorithms to the BCC dataset. The RF classifier has been utilized, and its hyperparameters have been optimized using the Optuna framework. Three wrapper-based feature selection techniques—SFS, SBS, and EFS—have been applied to identify the most relevant features. Among these methods, SFS and EFS selected the same feature subset and achieved identical performance, with accuracy of 85.71%, F1-score of 83.87%, precision of 92.85%, and recall of 76.47%, while SBS showed comparatively lower performance. The feature subsets obtained from the three wrapper methods were also combined to construct two ensemble voting classifiers using soft and hard voting strategies. Evaluation showed that hard voting achieved better performance, reaching an accuracy of 85.71%, F1-score of 83.87%, precision of 92.85%, and recall of 76.47%, whereas soft voting achieved an accuracy of 80.00%, F1-score of 77.42%, precision of 85.71%, and recall of 70.59%. Finally, DiCE analysis of misclassified cases showed BMI and Glucose to be the key features for correct BC prediction, with HOMA, Adiponectin, and Resistin having a smaller influence.

The RF classifier model achieved adequate results in the present study; however, the BCC dataset is small, and small datasets often do not represent the full variability of the problem. In this research, we used the Optuna technique to optimize hyperparameters for the RF classifier and utilized a wrapper-based feature selection technique. In addition, an ensemble voting classifier was applied. This technique was applied to the BCC dataset, representing the first such work to employ an RF classifier model.

To improve generalizability, future research could use an RF classifier or other classifiers such as XGBoost, LightGBM, CatBoost, etc. Every classifier is dataset dependent, which indicates that the results will vary according to the model’s performance. In addition, other wrapper-based techniques such as the Genetic Algorithm (GA), Bird Swarm Algorithm (BSA), Whale Optimization Algorithm (WOA), etc., can be applied. This study has several limitations. First, it used a small publicly available dataset, which may limit generalizability. Second, it focused on interpretability using DiCE, without comparing it to other methods such as SHAP or LIME. This topic will be reserved for future work.

## Figures and Tables

**Table 1:** Breast Cancer Coimbra dataset feature description.

Index	Feature Name	Data Type	Value Range (Min–Max)
0	Age ($f_{1}$)	Numerical	24–86 (Years)
1	BMI ($f_{2}$)	Numerical	18.370–38.579 (kg/m^2^)
2	Glucose ($f_{3}$)	Numerical	60–201 (mg/dL)
3	Insulin ($f_{4}$)	Numerical	2.432–58.460 (μU/mL)
4	HOMA ($f_{5}$)	Numerical	0.467–25.050
5	Leptin ($f_{6}$)	Numerical	4.311–90.280 (ng/mL)
6	Adiponectin ($f_{7}$)	Numerical	1.656–38.040 (μg/mL)
7	Resistin ($f_{8}$)	Numerical	3.210–82.100 (ng/mL)
8	MCP.1 ($f_{9}$)	Numerical	45.843–1698.440 (pg/dL)
9	Classification ($f_{10}$)	Binary	0 or 1

**Table 2:** Hyperparameter range and description.

Hyperparameter	Type	Range	Description
n_estimators	Integer	50–300	Number of estimator to build for the ensemble
min_group	Integer	2–5	Represents minimum size of an subsample group
max_group	Integer	min_group–5	Represents maximum size of an subsample group
remove_proportion	Float	0.1–0.7	Represents proportion of cases to be removed per group.

**Table 3:** Best hyperparameter values for classifiers.

n_estimators	min_group	max_group	remove_proportion
144	5	5	0.5391963650868431

**Table 4:** Performance comparison of SFS, SBS, EFS, and 5-fold cross-validation (mean result) using Rotation Forest classifier.

Aspect	SFS/EFS	SBS	5-Fold Cross-Validation(Mean Result)
Classifier	Rotation Forest	Rotation Forest	Rotation Forest
Accuracy (%)	85.71	68.57	73.99
F1 Score (%)	83.87	68.57	67.34
Precision (%)	92.85	66.66	70.48
Recall (%)	76.47	70.58	68.11
Best Features	Age, BMI, Glucose, Adiponectin, Resistin	Age, Glucose, HOMA, Adiponectin, Resistin	All

**Table 5:** Performance comparison of soft and hard voting ensemble methods.

Voting Method	Accuracy %	F1-Score %	Precision %	Recall %
Soft Voting (Train)	100	100	100	100
Hard Voting (Train)	100	100	100	100
Soft Voting (Test)	80.00	77.42	85.71	70.59
Hard Voting (Test)	85.71	83.87	92.86	76.47

**Table 6:** Misclassified Index (Idx) under Soft Voting (SV) and Hard Voting (HV).

Attribute	Idx 18	Idx 24	Idx 26	Idx 30	Idx 31	Idx 100	Idx 114
Voting Type	SV/HV	SV/HV	SV	Soft/Hard	SV/HV	SV	SV/HV
Age	64	54	50	66	53	74	72
BMI	34.53	30.48	38.58	36.21	36.79	28.65	25.59
Glucose	95	90	106	101	101	88	82
HOMA	1.04	1.23	1.75	3.87	2.53	0.65	0.57
Adiponectin	5.46	9.73	20.03	7.54	20.03	7.65	33.75
Resistin	6.70	10.19	11.78	22.32	10.26	18.36	3.27
Label Pair	1 → 2	1 → 2	1 → 2	1 → 2	1 → 2	2 → 1	2 → 1

**Table 7:** Comparison of our work with other models.

Model Name	Accuracy %	F1 Score %	Precision %	Recall %
XGBoost	82.86	83.33	83.33	83.33
LightGBM	80.00	78.79	81.25	76.47
CatBoost	77.14	75.00	80.00	70.59
Our Work-15-Fold Cross-Validation (RF)	75.43	71.61	75.00	70.00
Our Work-2SFS/EFS (RF)	85.71	83.87	92.86	76.47
Our Work-2SBS (RF)	68.57	68.57	66.66	70.58
Our Work-3Ensemble Voting Classifier (Soft Voting)	80.00	77.42	85.71	70.59
Our Work-3Ensemble Voting Classifier (Hard Voting)	85.71	83.87	92.86	76.47

**Table 8:** Counterfactual explanations for SV model (misclassified Index 26 and Index 100).

Index	Age	BMI	Glucose	HOMA	Adiponectin	Resistin	Class
Original Instance (Predicted: 2, Actual: 1)							
26	50	38.58	106	1.75	4.67	11.78	2
Counterfactuals (New Predicted Class: 1)							
1	–	–	89.0	–	–	58.17	1
2	–	32.32	76.0	–	–	–	1
3	–	68.0	–	–	7.54	–	1
Original Instance (Predicted: 1, Actual: 2)							
100	74	28.65	88	0.65	7.65	18.36	1
Counterfactuals (New Predicted Class: 2)							
1	–	–	–	14.98	–	49.78	2
2	–	–	–	15.50	23.90	–	2
3	–	29.89	189.0	–	–	–	2

**Table 9:** Counterfactual explanations for both SV and HV models (misclassified Index 24 and Index 31).

Index	Age	BMI	Glucose	HOMA	Adiponectin	Resistin	Class
Original Instance (Predicted: 2, Actual: 1)							
24	54	30.48	90.0	1.23	9.73	10.19	2
Counterfactuals (New Predicted Class: 1)							
1	-	35.81	-	-	33.06	-	1
2	-	36.49	-	-	-	-	1
3	-	28.86	69.0	-	-	-	1
Original Instance (Predicted: 2, Actual: 1)							
31	53	36.79	101.0	2.53	20.03	10.26	2
Counterfactuals (New Predicted Class: 1)							
1	-	-	66.0	-	-	-	1
2	-	-	81.0	-	-	8.78	1
3	-	-	78.0	14.09	-	-	1

**Table 10:** Counterfactual explanations for both SV and HV models (correctly predicted Index 1 and Index 52).

Index	Age	BMI	Glucose	HOMA	Adiponectin	Resistin	Class
Original Instance (Actual: 1, Predicted: 1)							
1	83	20.69	92	0.70	5.42	4.06	1
Counterfactuals (New Predicted Class: 2)							
1	-	-	149.0	-	-	28.45	2
2	-	-	-	15.60	-	51.79	2
3	-	-	162.0	-	-	-	2
Original Instance (Actual: 2, Predicted: 2)							
52	45	21.30	102	3.48	21.05	23.03	2
Counterfactuals (New Predicted Class: 1)							
1	-	-	60.0	-	-	-	1
2	-	-	77.0	-	36.92	-	1
3	-	-	81.0	-	-	8.77	1

## Data Availability

The dataset used in this study is publicly available and can be accessed from the following URL: https://archive.ics.uci.edu/dataset/451/breast+cancer+coimbra (accessed on 25 April 2025). The code used for this study is available from the corresponding author upon reasonable request.

## References

[1] Lakhani S.R., Ellis I.O., Schnitt S.J., Tan P.H., van de Vijver M.J. (2012). WHO Classification of Tumours of the Breast.

[2] World Health Organization (WHO) Breast Cancer. https://www.who.int/news-room/fact-sheets/detail/breast-cancer.

[3] Silva S.N., Gomes B.C., Andre S., Felix A., Rodrigues A.S., Rueff J. (2021). Male and female breast cancer: The two faces of the same genetic susceptibility coin. Breast Cancer Res. Treat..

[4] Bray F., Laversanne M., Sung H., Ferlay J., Siegel R.L., Soerjomataram I., Jemal A. (2024). Global cancer statistics 2022: GLOBOCAN estimates of incidence and mortality worldwide for 36 cancers in 185 countries. CA A Cancer J. Clin..

[5] World Cancer Research Fund International (2024). Breast Cancer Statistics. https://www.wcrf.org/preventing-cancer/cancer-statistics/breast-cancer-statistics/.

[6] Assegie T.A. (2021). An optimized K-Nearest Neighbor based breast cancer detection. J. Robot. Control JRC.

[7] Patrício I., Pereira M.A., Crisóstomo J., Matafome A., Gomes R., Seiça J., Ferreira P.B. (2018). Using Resistin, glucose, age and BMI to predict the presence of breast cancer. BMC Cancer.

[8] Polat K., Sentürk U. A novel ML approach to prediction of breast cancer: Combining of mad normalization, KMC based feature weighting and AdaBoostM1 classifier. Proceedings of the 2018 2nd International Symposium on Multidisciplinary Studies and Innovative Technologies (ISMSIT).

[9] Li Y., Chen Z. (2018). Performance evaluation of machine learning methods for breast cancer prediction. Appl. Comput. Math..

[10] Islam M.M., Poly T.N. (2019). Machine learning models of breast cancer risk prediction. bioRxiv.

[11] Abdulkareem A.H., Kasapbaşı M.C. (2020). Enhancing detection method of breast cancer using Coimbra dataset. İstanbul Ticaret Üniversitesi Teknol. Uygulamalı Bilim. Derg..

[12] Akiba T., Sano S., Yanase T., Ohta T., Koyama M. Optuna: A next-generation hyperparameter optimization framework. Proceedings of the 25th ACM SIGKDD International Conference on Knowledge Discovery & Data Mining.

[13] Bergstra J., Bardenet R., Bengio Y., Kégl B. (2011). Algorithms for hyper-parameter optimization. Advances in Neural Information Processing Systems.

[14] Rodriguez J.J., Kuncheva L.I., Alonso C.J. (2006). Rotation forest: A new classifier ensemble method. IEEE Trans. Pattern Anal. Mach. Intell..

[15] Sktime Developers Sktime.Classification.Sklearn.RotationForest—Sktime Documentation. https://www.sktime.net/en/stable/api_reference/auto_generated/sktime.classification.sklearn.RotationForest.html.

[16] Ververidis D., Kotropoulos C. Sequential forward feature selection with low computational cost. Proceedings of the 2005 13th European Signal Processing Conference.

[17] Yıldırım A.A., Özdoğan C., Watson D. (2016). Parallel data reduction techniques for big datasets. Big Data: Concepts, Methodologies, Tools, and Applications.

[18] Guyon I., Elisseeff A. (2003). An introduction to variable and feature selection. J. Mach. Learn. Res..

[19] Nersisyan S., Novosad V., Galatenko A., Sokolov A., Bokov G., Konovalov A., Alekseev D., Tonevitsky A. (2022). ExhauFS: Exhaustive search-based feature selection for classification and survival regression. PeerJ.

[20] Mothilal R.K., Sharma A., Tan C. Explaining machine learning classifiers through diverse counterfactual explanations. Proceedings of the 2020 Conference on Fairness, Accountability, and Transparency.

[21] Muti P., Quattrin T., Grant B.J., Krogh V., Micheli A., Schünemann H.J., Ram M., Freudenheim J.L., Sieri S., Trevisan M. (2002). Fasting glucose is a risk factor for breast cancer: A prospective study. Cancer Epidemiol. Biomarkers Prev..

[22] Sun S., Sun Y., Rong X., Bai L. (2019). High glucose promotes breast cancer proliferation and metastasis by impairing angiotensinogen expression. Biosci. Rep..

[23] Hou Y., Zhou M., Xie J., Chao P., Feng Q., Wu J. (2017). High glucose levels promote the proliferation of breast cancer cells through GTPases. Breast Cancer: Targets and Therapy.

[24] Avtanski D., Garcia A., Caraballo B., Thangeswaran P., Marin S., Bianco J., Lavi A., Poretsky L. (2019). Resistin induces breast cancer cells epithelial to mesenchymal transition (EMT) and stemness through both adenylyl cyclase-associated protein 1 (CAP1)-dependent and CAP1-independent mechanisms. Cytokine.

[25] Kang J.-H., Yu B.-Y., Youn D.-S. (2007). Relationship of serum adiponectin and resistin levels with breast cancer risk. J. Korean Med. Sci..

[26] Assiri A.M.A., Kamel H.F.M., Hassanien M.F.R. (2015). Resistin, visfatin, adiponectin, and leptin: Risk of breast cancer in pre- and postmenopausal Saudi females and their possible diagnostic and predictive implications as novel biomarkers. Dis. Markers.

[27] Mantzoros C., Petridou E., Dessypris N., Chavelas C., Dalamaga M., Alexe D.M., Papadiamantis Y., Markopoulos C., Spanos E., Chrousos G. (2004). Adiponectin and breast cancer risk. J. Clin. Endocrinol. Metab..

[28] Tzenios N., Tazanios M.E., Chahine M. (2024). The impact of BMI on breast cancer–an updated systematic review and meta-analysis. Medicine.

